# A selective detection of lysophosphatidylcholine in dried blood spots for diagnosis of adrenoleukodystrophy by LC-MS/MS

**DOI:** 10.1016/j.ymgmr.2016.02.007

**Published:** 2016-03-18

**Authors:** Ryuichi Mashima, Misa Tanaka, Eri Sakai, Hidenori Nakajima, Tadayuki Kumagai, Motomichi Kosuga, Torayuki Okuyama

**Affiliations:** a Department of Clinical Laboratory Medicine, National Center for Child Health and Development, 2-10-1 Okura, Setagaya-ku, Tokyo 157-8535, Japan; b Research Institute, National Center for Child Health and Development, 2-10-1 Okura, Setagaya-ku, Tokyo 157-8535, Japan; c Division of Medical Genetics, National Center for Child Health and Development, 2-10-1 Okura, Setagaya-ku, Tokyo 157-8535, Japan

**Keywords:** Adrenoleukodystrophy, LC-MS/MS, DBS

## Abstract

X-linked adrenoleukodystrophy (X-ALD) is a rare inherited metabolic disorder characterized by an impaired beta-oxidation of very long chain fatty acids in the peroxisomes. Recent studies have suggested that 1-hexacosanoyl-2-hydroxy-*sn*-glycero-3-phosphocholine (Lyso-PC 26:0) can be a sensitive biomarker for X-ALD. Although approximately 10-fold increase in the concentration of Lyso-PC 26:0 in DBSs from X-ALD-affected individuals were reported, whether the carriers might be distinguished from the healthy controls remained unclear. To address this question, we have validated previously developed LC-MS/MS-based analytical procedures using QC DBS. We found that the recovery of Lyso-PC 26:0 from the QC DBSs was 73.6 ± 0.3% when 2 μM of Lyso-PC 26:0 was spiked into the blood. Based on this result, the amounts of Lyso-PC 26:0 in the controls and ALD-affected individuals were 0.090 ± 0.004 (*n* = 11) and 1.078 ± 0.217 (*n* = 4) pmol/DBS, respectively. Interestingly, the concentration of Lyso-PC 26:0 in the carriers were 0.548 ± 0.095 pmol/DBS (*n* = 3), indicating that the carriers and the healthy controls can be distinguished. These results suggest that LC-MS/MS-based technique can be used for the detection of asymptomatic carriers and X-ALD-affected subjects in the newborn screening.

## 1 Introduction

X-linked adrenoleukodystrophy (X-ALD) is a rare inherited metabolic disorder characterized by an impaired beta-oxidation of very long chain fatty acids (VLCFAs) in the peroxisomes [1]. X-ALD is caused by mutations in the *ABCD1* gene encoding a peroxisomal ABC transporter ALDP. Phenotypically, X-ALD shows a variety of clinical manifestations. One major form involves childhood cerebral ALD presenting as progressive inflammatory demyelination in the brain in males, leading to rapid cognitive and neurological decline. Another form of X-ALD, known as adrenomyeloneuropathy, is characterized by a slowly progressive axonopathy that usually appears between 20 and 30 years of age in males. In contrast, affected females may develop milder symptoms of adrenomyeloneuropathy, at late in life [2]. As curative therapy, hematopoietic stem cell therapy has been performed, however its effectiveness is limited in the earliest stage of the onset of cerebral ALD [3]. In addition, a clinical study of gene therapy that transfers wild-type *ABCD1* gene to hematopoietic stem cells using a lentiviral vector *ex vivo* shows promising results [4].

A hallmark of X-ALD is an accumulation of VLCFAs in the body [5]. Earlier studies determined plasma VLCFAs as their methyl esters followed by detection using GC or GC-MS assays [5], [6]. Subsequently, among many species of VLCFAs, 1-hexacosanoyl-2-hydroxy-*sn*-glycero-3-phosphocholine (Lyso-PC 26:0) has been proposed as a sensitive biomarker for X-ALD [7], [8], [9], [10]. The milestone study demonstrated that an increasing Lyso-PC 26:0 levels in the dried blood spots (DBSs) from X-ALD patients using liquid chromatography with mass spectrometric detection (LC-MS/MS) [7]. Subsequent studies similarly demonstrated the elevation of Lyso-PC 26:0 in DBS from affected patients using a high-throughput assay with MS/MS detection [9], [10], [11], [12]. Recent studies showed that Lyso-PC 26:0 can be simultaneously extracted from a DBS with acylcarnitine, indicating that Lyso-PC 26:0 can also be analyzed using the same methanolic extracts for amino acid measurement that is widely employed in newborn screenings [10], [13]. Thus, all of these studies show that Lyso-PC 26:0 is an established sensitive biomarker for X-ALD. However, whether the carriers might be detectable by Lyso-PC 26:0 as a biomarkers remains unclear. Thus, this study aims to explore whether the carriers might be distinguished from the healthy controls.

## 2 Experimental procedures

### 2.1 Materials

All Lyso-PC standards were purchased from Avanti Polar Lipids (Alabaster, Alabama). Acetonitrile and methanol were purchased from Fischer Scientific (Tokyo, Japan). Isopropanol was purchased from Wako Pure Chemicals (Tokyo, Japan). Deionized water was obtained from a Milli-Q water system (Millipore, Milford, MA). Ammonium acetate and formic acid were purchased from Kanto Chemical (Tokyo, Japan). The other reagents used in this study were of the highest grade commercially available. CDC-validated QC DBSs were received from Dr Christopher A. Haynes (Newborn Screening and Molecular Biology Branch, Centers for Disease Control and Prevention, Atlanta, GA) [14].

### 2.2 Preparation of DBS

DBS for calibration, recovery, and precision studies were prepared as follows: whole blood was collected in an EDTA-containing vacutainer and then spiked with 2 μM of methanolic Lyso-PC 26:0; an aliquot (50 μl) of spiked blood was subsequently spotted on filter paper that is specifically manufactured for medical diagnostic use (Advantec, Tokyo, Japan) and air-dried overnight at room temperature; these DBS were finally stored at − 20 °C in zip-lock bags with a desiccant prior to analysis. In this study, we estimated that a 3 mm DBS punch contains 3.3 μl of whole blood.

### 2.3 Extraction

A single 3 mm disc was punched from the DBS and transferred into a 1.5 ml Eppendorf tube. Methanol (100 μl) containing an internal standard D_4_-Lyso-PC 26:0 (100 nM) was added to each tube and blood lipids were extracted by sonication for 15 min at 4 °C. Then, the eluates were centrifuged at 15,000 rpm for 10 min at 4 °C, followed by filtration using a Cosmospin Filter G (pore size: 0.22 μm, Nacalai Tesque, Kyoto, Japan). This extraction step was repeated twice. After the final spin filtration, all of the eluates were dried and then reconstituted with 100 μl of methanol/5 mM ammonium acetate (90/10). Finally, the solubilized samples were transferred to vials for the autosampler.

### 2.4 LC-MS/MS

Blood lipids extracted from the DBS were separated on a GL Sciences InertSustain C18 column (2.1 × 100 mm, 3 μm) with gradient elution from 5 mM of ammonium acetate to 0.1% formic acid in methanol over 10 min at a flow rate of 0.2 ml/min. A volume of 10 μl of the sample was injected for LC-MS/MS. Lyso-PC 26:0 was detected on an LCMS-8040 mass spectrometer (Shimadzu, Kyoto, Japan) by ESI-positive mode equipped with an Nexera MP UHPLC and an SIL-30AC autosampler (Shimadzu). The data were then analyzed using LabSolutions data analysis software (Shimadzu).

### 2.5 Statistics

The data are expressed as mean ± SEM as indicated in the figure legends. The statistical significance of differences in the mean values from the two groups was determined by a Student's *t*-test. A difference of *P* < 0.05 was considered significant.

## 3 Results and discussion

To ask whether the accumulating Lyso-PC 26:0 in DBS from ALD-affected individuals can be measured properly, we prepared in-house generated QC DBSs that was supplemented with 6.6 pmol Lyso-PC 26:0 per a 3-mm DBS (i.e., 2 μM of Lyso-PC 26:0 in the blood). The result of recovery experiment revealed that 4.95 ± 0.02 pmol/DBS of Lyso-PC 26:0 was recovered from the spiked DBS, demonstrating that 73.6 ± 0.3% of spiked Lyso-PC 26:0 was detected (Table 1). Under these experimental conditions, there was a linear response of Lyso-PC 26:0 using the DBS distributed by CDC (1.0 and 5.0 μM of Lyso-PC 26:0 in the blood) (Supplementary Fig. 1).

**Fig. 1:**
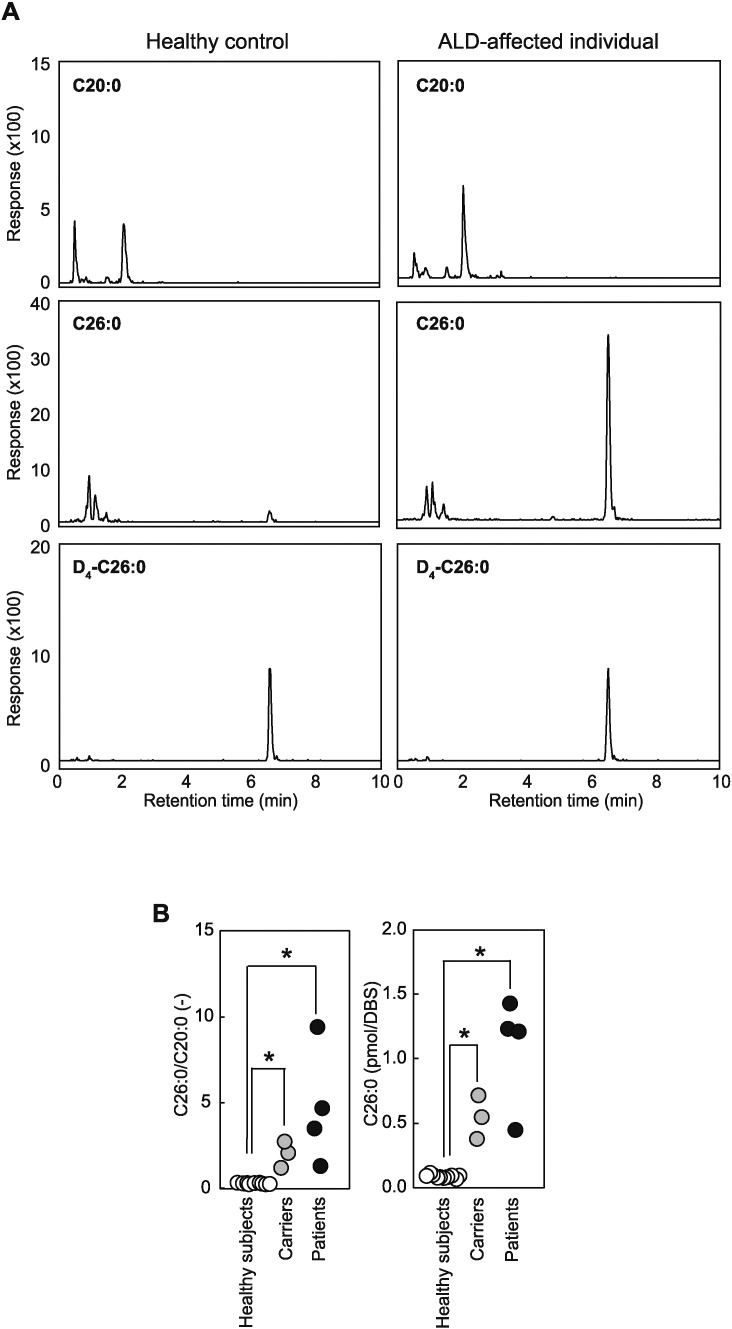
An accumulation of Lyso-PC 26:0 in ALD patients and carriers. (A) Representative chromatograms obtained from a healthy control (left) or an ALD-affected individual (right). Lyso-PC 20:0, Lyso-PC 26:0 and D_4_-Lyso-PC 26:0 in the lipid extracts from DBS from a healthy control or an ALD-affected individual were subjected to analysis using LC-MS/MS method described in the Experimental Procedures. (B) Comparison of the ratios of Lyso-PC 26:0 to Lyso-PC 20:0 (C26:0/C20:0) and the amounts of Lyso-PC 26:0 (pmol/DBS). The levels of Lyso-PC 26:0 and Lyso-PC 20:0 were determined and their ratios in healthy subjects (white, *n* = 11), carriers (gray, *n* = 3), and ALD patients (black, *n* = 4) were presented. Data expressed as mean ± SEM. * indicates *P* < 0.05.

We further employed this validated method to evaluate the levels of Lyso-PC 26:0 in the methanolic lipid extracts from the DBS of ALD patients. As shown in Fig. 1A Top, Lyso-PC 20:0, an endogenously present analog of Lyso-PC 26:0, was detected at nearly similar levels in both healthy subjects and ALD patients. However, there was a marked accumulation of Lyso-PC 26:0 in the lipids extracted from the DBS of ALD patients compared to those of healthy subjects (Fig. 1A Middle), while the amount of the internal standard D_4_-Lyso-PC 26:0 remained unchanged (Fig. 1A Bottom). The ratio of Lyso-PC 26:0 to Lyso-PC 20:0 (C26:0/C20:0), another widely used measure for the diagnosis of ALD-affected subjects, was also consistently higher in the patients compared to healthy subjects with a statistically significant difference (Fig. 1B, left). Consistently, the level of Lyso-PC 26:0 (pmol/DBS) in the ALD-affected patients was also elevated (Fig. 1B, right), demonstrating that these two measures could be used for the diagnosis of ALD as previously reported. Although earlier studies have suggested the accumulation of C26:0 of the carriers in the plasma by GC [5], there was no evidences that Lyso-PC 26:0 in the blood behave similarly. To ask this question, we analyzed the DBS from the carriers. As shown in Fig. 1B, the values of them were significantly elevated compared to those of healthy subjects, whereas there was no difference of both C26:0/C20:0 (-) and Lyso-PC 26:0 (pmol/DBS) between the carriers and ALD-affected patients.

There is an increasing demand for the newborn screening for X-ALD, because cerebral ALD is curable by hematopoietic stem cell transplantation when the affected individuals are found at asymptomatic conditions [3]. Thus, New York State commenced NBS for X-ALD in 2013 [15]. At the same time, CDC began to distribute the QC DBS for Lyso-PC 26:0 for X-ALD [14]. When NBS for ALD become available, there is a concern whether only males or males plus females are to be screened. As reported, female carriers are less symptomatic and only limited individuals show less severe disease phenotype at the late of life [2]. From practical point of view, we might not be able to identify whether the subject is male or female from the description on the DBS, because the gender of the baby is not usually disclosed on the DBS in Japan. Under these circumstances, the positive results of females will be informed to the parents. Whether this is beneficial for the family of the carriers has not been addressed.

X-ALD is one of the peroxisomal disorders that involve Zellweger syndrome, Refsum disease, or other defects of single enzyme defects. Refsum disease causes an accumulation of branched fatty acid such as phytanic acid, therefore this disease can be easily distinguished from X-ALD [16]. Zellweger syndrome-affected individuals normally exhibit extremely high accumulation of C26:0 and a loss of plasmalogen at the same time [15], [17]. Other single enzyme defects of peroxisomal fatty acid oxidation other than ALD also show an elevating plasma C26:0, therefore genomic data using next generation sequencing may be required to distinguish them individually [6], [15]. In any cases, the accumulation of Lyso-PC 26:0 can be a sensitive biomarker for peroxisomal disorders including X-ALD.

Conclusively, we provided evidence that the carriers of X-ALD show an increasing Lyso-PC 26:0 in the blood compared to the healthy controls. Further large scale study will elucidate the ratio of false-positives in the carriers using this assay.

The following is the supplementary data related to this article.

Supplementary Fig. 1 Dose response curve of Lyso-PC 26:0 in the CDC QC DBS by LC-8040. Supplementary Fig. 1

## Figures and Tables

**Table 1:** Recovery of Lyso-PC species from the spiked blood spot specimens.

Additive	Amount	Detected		Recovery^a^	
Lyso-PC 20:0	Lyso-PC 26:0	Lyso-PC 20:0	Lyso-PC 26:0
(pmol/DBS)	(pmol/DBS)	(pmol/DBS)	(%)	(%)
None	NA^b^	0.45 ± 0.01	0.10 ± 0.00	NA	NA
Lyso-PC 20:0	6.6	4.32 ± 0.03	0.11 ± 0.00	58.8 ± 0.4	NA
Lyso-PC 26:0	6.6	0.57 ± 0.00	4.95 ± 0.02	NA	73.6 ± 0.3

Data were expressed as mean ± SEM (*n* = 5–7).

a Recovery (%) is defined as (the amount of detected Lyso-PC – the amount of endogenous Lyso-PC)/the amount of added Lyso-PC × 100.

b NA, not applicable.
