# Supplementary figures and images for: A selective detection of lysophosphatidylcholine in dried blood spots for diagnosis of adrenoleukodystrophy by LC-MS/MS
Source: Mol Genet Metab Rep. 2016 Mar 18;7:16–9. doi: 10.1016/j.ymgmr.2016.02.007 (PMC4908058; doi:10.1016/j.ymgmr.2016.02.007)

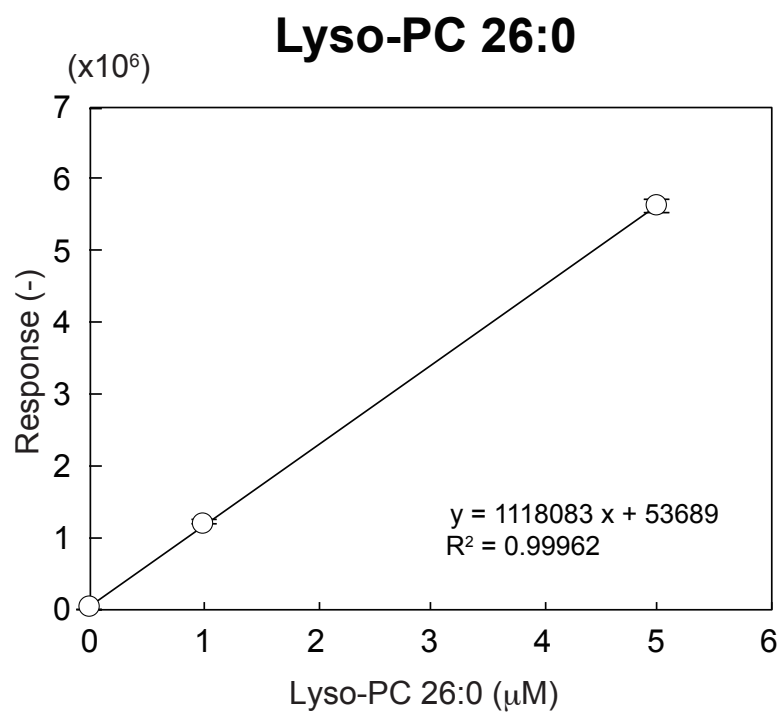

Supplementary Fig. 1

Dose response curve of Lyso-PC 26:0 in the CDC QC DBS by LC-8040.

Supplement: Supplementary Fig. 1 — Dose response curve of Lyso-PC 26:0 in the CDC QC DBS by LC-8040. [file mmc1.pdf]
